# Beyond Feasibility: Critical Steps Toward Drone-based Organ Transport

**DOI:** 10.1097/TP.0000000000005527

**Published:** 2025-09-23

**Authors:** Robson G. Gilmour, Mekhola Hoff

**Affiliations:** 1 Edinburgh Medical School, University of Edinburgh, Edinburgh, United Kingdom.; 2 Royal Infirmary of Edinburgh, Edinburgh Transplant Centre, Edinburgh, United Kingdom.

## Abstract

**Figure:**
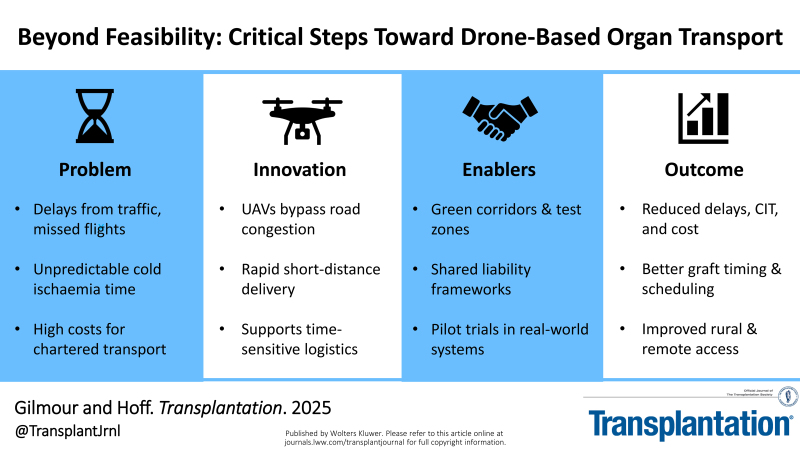

## INTRODUCTION

Drones, or uncrewed aerial vehicles (UAVs), are becoming increasingly visible in healthcare logistics. In several countries, they already deliver blood, vaccines, and laboratory samples to remote areas, helping overcome geographic and infrastructure challenges. Trials in high-income countries also show that UAVs can deliver medicines faster and with lower emissions than conventional transport methods. Globally, transplant systems face common logistical challenges, particularly around time-critical organ delivery.

Transplantation presents a powerful case for innovation. Organ transplant success depends not only on matching but on timing. Once retrieved, an organ must be transplanted within a narrow window, known as the cold ischemia time. Delays reduce viability and worsen outcomes.^1^ Although ambulances, aircraft, and couriers generally work well, they are costly, complex to coordinate, and vulnerable to delays. A single miscommunication can result in an organ no longer usable.

Drones offer a potential way to reduce these delays by replacing one leg of a journey or streamlining local transfers. So far, a few successful test flights have been reported, but routine adoption requires more than just technical success. Regulatory clarity, cost-effectiveness, clinical evidence, and integration into existing systems must follow.

## CLINICAL PRIORITIES: FROM PROOF-OF-CONCEPT TO VALUE

Only 2 drone-based organ deliveries have been formally published: a 2019 kidney delivery in Baltimore and a 2021 lung transport in Toronto.^2,3^ Both involved the full journey from donor to recipient hospital and were preceded by extensive trial runs—47 and >400, respectively. The kidney recipient was followed for 30 d posttransplant, and the lung recipient had an uneventful routine postoperative course, with no reported complications attributable to drone transport or otherwise in either case. However, both flights were covered under 5 km, and timing data, including loading and coordination logistics, were not clearly reported. These one-off, orchestrated missions confirm feasibility but could be considered to fall short of demonstrating real-world value.

Outstanding questions remain: How do UAVs perform over longer distances, in poor weather, or when transporting fragile grafts? A reduction from 60–90 min to 15 min may seem modest, but every minute counts—each additional hour of cold ischemia significantly increases graft failure and mortality.^4^ Beyond speed, UAVs could streamline workflows through automated packaging, digital labeling, and real-time documentation. These advantages may be especially relevant in rural or traffic-congested regions where conventional transport is less predictable. The value of UAVs may lie not only in faster delivery but also in supporting more responsive and integrated logistics.

However, most UAVs currently available for civilian use face significant limitations in range, speed, and payload capacity. Unlike military-grade models, commercially available drones typically carry only 5–20 kg and have operational ranges <50 km. These constraints restrict their use to short-distance missions and emphasize the importance of identifying realistic, high-impact applications before attempting to extend UAV roles more broadly.

One of the most immediately scalable opportunities lies in transporting time-sensitive blood and tissue samples, such as wet crossmatch material, HLA typing, or posttransplant biopsies. These are lighter, more compact, and face fewer regulatory barriers than organs—yet remain critical to transplant decisions and monitoring. UAV transport could help reduce delays in getting these samples to 24/7 laboratories, particularly outside working hours or across remote areas. As a lower-risk entry point, this use case may provide a commercially viable pathway to integrate UAVs into transplant workflows, offering operational learnings applicable to full organ transport in the future.

To move beyond feasibility and demonstrate real-world clinical value, future research must rigorously assess how UAV transport compares with existing logistics. Comparative trials should evaluate not only timing and temperature control but also mechanical stressors, such as vibration and torque, while tracking key clinical outcomes such as graft function, length of hospital stay, and overall survival. Simulation modeling, including digital twins and predictive analytics, can help anticipate risk under diverse conditions, such as poor weather, altitude changes, and airspace congestion. Meanwhile, pilot studies using relatively robust organs, such as kidneys, should be embedded within existing workflows on short, time-sensitive routes. These studies should assess not only safety but also responsiveness to unpredictable changes in transplant schedules, such as last-minute retrieval updates that currently require costly, chartered alternatives.

The goal must now shift from feasibility to value, demonstrating, in specific and measurable terms, that drones improve safety, efficiency, or clinical outcomes. For hospitals, this could mean lower costs and freed-up staff; for coordinators, more predictable handovers; for patients, better-preserved grafts and improved outcomes.

## REGULATORY GAPS: ENABLING MEDICAL UAV PATHWAYS

In most countries, drones flying beyond visual line of sight (BVLOS) are subject to strict regulation, with few provisions for emergency medical use. This limits UAV deployment in time-critical scenarios such as organ transport, where speed and predictability are vital.

A particularly significant challenge arises in major metropolitan areas, where controlled and congested airspace severely limits UAV operations. This includes, for example, restricted zones in cities such as London, New York, and Dubai, which are home to major transplant centers, where UAV access is tightly constrained because of dense commercial aviation and strict air traffic control. In such environments, drones are often prohibited or require complex, time-consuming approvals, and permissions for BVLOS operations are rarely granted. As a result, UAV deployment is most restricted in exactly the locations where faster, more flexible transport could offer the greatest clinical benefit.

To overcome these limitations, purposeful regulatory innovation is needed. One key proposal is the creation of dedicated “green corridor” airspace classifications for emergency medical drones. These would be preapproved, low-interference routes with real-time oversight that allow BVLOS operations during urgent missions. Although “green corridor” may imply rural airspace, here it refers to designated and controlled airspace, approved for UAV passage even over built environments such as roads, housing, or hospital campuses. These routes can be developed to ensure safety and compliance without requiring major ground infrastructure or disruption. In parallel, governments should consider fast-track approvals or conditional exemptions for organ retrieval flights, recognizing their urgency and public health value. National test zones, where medical UAVs can be trialed under regulatory supervision, will also be essential to safely refine and validate these pathways.

Promising models already exist. In Rwanda, the Zipline programme uses government-sanctioned UAV corridors for routine medical deliveries. In the United Kingdom, the Care & Equity – Healthcare Logistics UAS Scotland and National Health Service (NHS)-Apian initiatives are actively designing integrated airspace frameworks for healthcare logistics. Internationally, coordination among agencies such as the Civil Aviation Authority, Federal Aviation Administration, and European Union Aviation Safety Agency will be crucial to ensuring UAV safety and interoperability across transplant networks that increasingly span borders.

## LEGAL AND ETHICAL CONSIDERATIONS

A major barrier to clinical adoption is the lack of defined legal responsibility in the event of a transport failure. In the United Kingdom, under the Human Tissue Authority (HTA) framework, transporting organs is a licensable activity,^5^ and any organization involved, including drone operators, must either hold an HTA license or operate under the authority of one. Comparable oversight structures exist elsewhere. In the United States, for example, the Food and Drug Administration and Health Resources and Services Administration regulate the safety of organ and tissue handling, whereas the United Network for Organ Sharing (UNOS) develops allocation policies. However, day-to-day allocation decisions and transport logistics are performed by regional Organ Procurement Organizations (OPOs) using UNOS-defined algorithms. Similarly, Eurotransplant and Scandiatransplant oversee allocation coordination across multinational systems with individual regulatory frameworks. Although the specifics vary, the need for clear accountability and licensing across jurisdictions remains universal.

Responsibility for safe transport is currently shared but not always explicitly defined. Under gift law, recipients have no legal right to an organ until it is transplanted, and transplant centers or OPOs are unlikely to be held liable unless gross negligence can be demonstrated. However, the use of UAVs introduces a new transport modality that could shift perceptions of acceptable risk. For instance, the failure of a drone-delivered organ—whether due to technical malfunction or process oversight—may raise questions about whether existing duty-of-care standards were met. This makes it essential that transplant centers, logistics providers, and drone operators collaboratively define clear standards of care, liability boundaries, and fallback protocols before UAV deployment becomes routine.

To that end, UAV-enabled organ transport must be underpinned by robust legal frameworks and clearly defined contractual relationships. Contracts between transplant centers, OPOs, and drone providers should specify liability, safety obligations, and protocols for reporting Serious Adverse Events and Reactions. Standard Operating Procedures must align with national regulatory expectations—such as those of the HTA—and should include chain-of-custody tracking, temperature monitoring, and documentation procedures. Oversight mechanisms, including audits and licensing reviews, will be critical to ensure compliance.

Several governance models may be worth exploring. Shared liability models—where responsibility is distributed between transplant centers, UAV providers, and national coordination bodies—could help mitigate uncertainty. Establishing minimum technical and operational standards for medical drone transport would also promote consistency across providers. Although most transplant systems do not currently require patients to consent to a specific mode of organ transport, offering transparency around UAV use—when it does not compromise confidentiality—may support public trust. National bodies such as NHS Blood and Transplant, UNOS, and Eurotransplant are well placed to develop coordinated guidance on the legal and ethical integration of UAVs.

Ultimately, public and professional confidence will depend on whether UAV transport can meet the same safety and ethical standards as existing methods. That means not only proving technical reliability but proactively defining legal frameworks that reflect the complexity of shared responsibility in modern transplant logistics.

## COST AND ENVIRONMENTAL MODELING

Air transport of organs can cost $7800–$15 000 per case in the United States.^6,7^ UAV trials suggest lower costs due to reduced fuel and labor. However, these were short, one-off flights and do not reflect the costs of routine use, including set-up for infrastructure and training.

Cost-effectiveness assessments must consider both initial investment and long-term savings. This includes modeling capital and operational costs over 50–100 flights annually, as well as infrastructure requirements such as drone landing zones, flight control centers, and specialized staff. These expenses must be weighed against potential savings, such as reduced reliance on ambulances or air charters, decreased coordination overhead, and improved transplant scheduling. Realistic economic modeling will provide transplant centers and policymakers with a clearer picture of where UAVs may be a financially sustainable option.

Environmental goals may also support UAV use. In a 2025 NHS pilot, drones replaced 4 van routes, cutting delivery times by 83% and CO_2_ emissions by 60%.^8^ However, full lifecycle assessments—including battery production, rare earth mineral extraction, end-of-life disposal, and potential noise pollution—are needed to demonstrate measurable impact. Comparative modeling with conventional transport methods, factoring in emissions, maintenance, and replacement cycles, will provide the robust evidence needed to support future policy decisions.

## SYSTEM INTEGRATION: IDENTIFYING THE RIGHT USE CASES

Drones are not intended to replace existing organ transport systems but to complement them, especially in cases where traditional methods are costly, delayed, or logistically inefficient.

**FIGURE 1.**
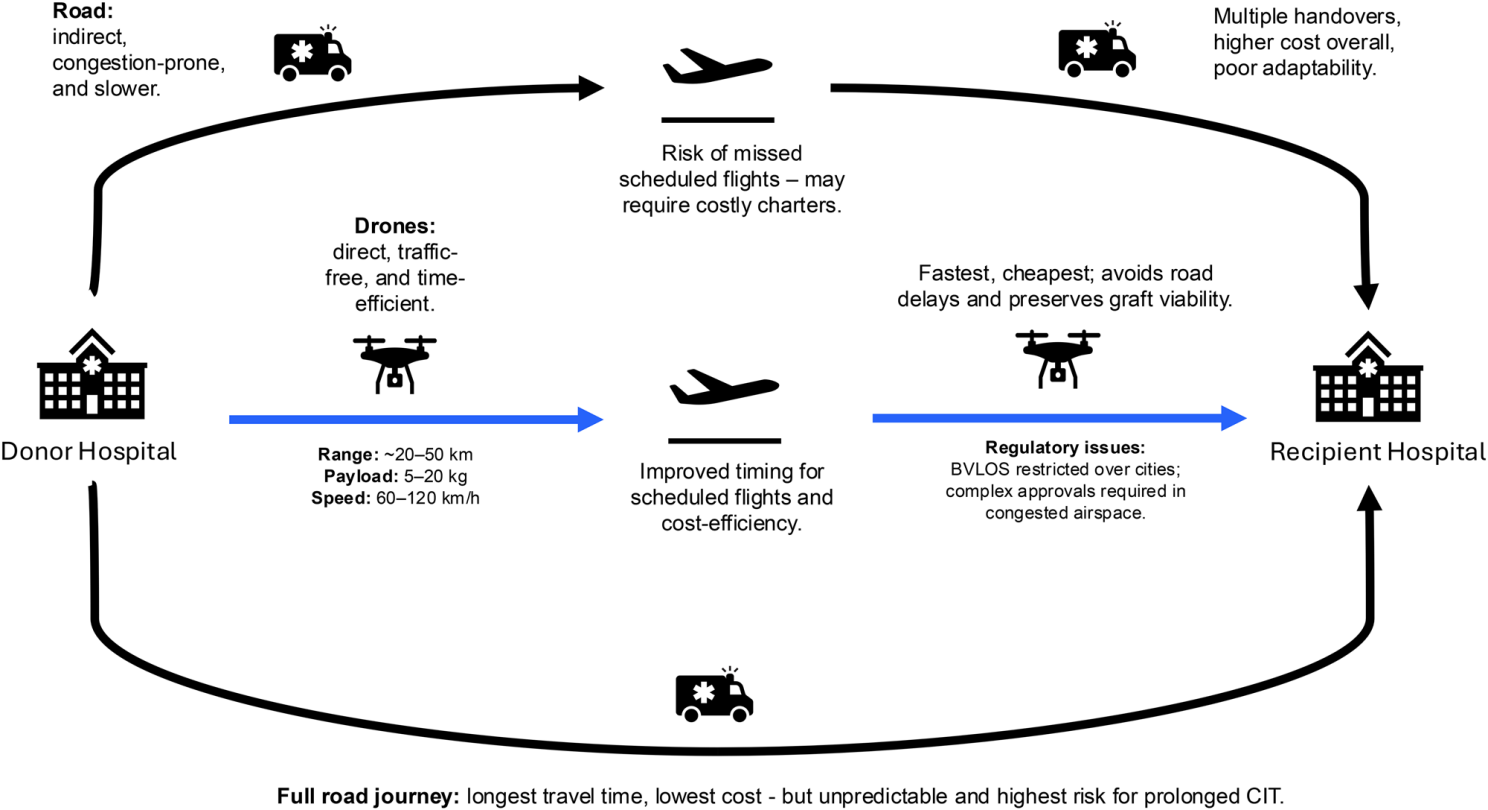
Schematic of UAV integration into organ transport pathways. Black arrows represent current transport methods (road–air–road or full road journeys), which are often associated with delays, high costs, and variable cold ischemia times—in some cases leading to organ decline. Blue arrows indicate proposed UAV-enhanced segments, where drones could replace either or both of the first and last legs of the journey, offering traffic-free delivery to reduce delays and preserve graft viability. Key technical specifications (range, payload, speed) and regulatory constraints (eg, BVLOS restrictions) are annotated. BVLOS, beyond visual line of sight; UAV, uncrewed aerial vehicle.

Instead, drones should be deployed strategically within existing systems. They may be most valuable in the early phase of logistics, transporting organs from the donor hospital to a central hub or airport, or in the final leg, delivering the organ from a regional airport to the recipient hospital. Intraregional transfers, particularly those between rural and urban centers, also represent a high-impact opportunity. These routes often suffer delays due to poor road infrastructure or traffic congestion, making them ideal candidates for UAV-enabled optimization (Figure 1).

Many final-leg transfers rely on ambulances, which are both costly and traffic-prone. UAVs could save both time and money on these short, predictable routes. In urban areas, drones may also help avoid delays from road closures, traffic jams, or vehicle accidents. Health systems should assess how many organ transfers occur within a 50 km radius—a range well suited to current drone capabilities. This applies not only in the United Kingdom but also in parts of the United States, Canada, and across Europe, where short-range transport often relies on congested road systems.

Seamless integration into existing transplant workflows will also require full digital compatibility. UAVs must support real-time tracking and dispatch systems, include chain-of-custody logging and continuous temperature monitoring, and interface effectively with the platforms already used by logistics teams and transplant coordinators. Without this level of compatibility, even the fastest UAV delivery may fall short of clinical expectations.

Pilot trials embedded within real-world transplant workflows—not isolated test flights—will be essential for identifying operational barriers, such as handovers, contamination control, real-time communication, and responsiveness to unexpected logistical changes.

## PUBLIC AND PROFESSIONAL READINESS

Public trust and professional support are essential to the adoption of drone-based organ delivery. A 2022 public dialogue in the United Kingdom found strong support for UAV use in healthcare—particularly in urgent scenarios such as organ transport—and also raised concerns about safety, regulation, and misuse.^9^

Transparent communication will be key. Public confidence depends on clear messaging and assurance that risks, such as equipment failure, severe weather, or interference, are actively managed.

Clinician engagement is equally vital. Transplant teams and coordinators should help design protocols, receive training, and contribute to ongoing evaluation to ensure UAV use supports clinical workflows rather than complicating them.

Hospitals must also prepare operational infrastructure, including designated landing zones, secure storage, and trained staff. These elements should be embedded into national transplant logistics standards to ensure safe and consistent implementation.

## CONCLUSION: A CRITICAL MOMENT FOR COORDINATION

Drone delivery in organ transplantation is no longer speculative—it is a real and promising option. However, real-world adoption will require coordinated international action, supported by strong governance, clinical evidence, and system-wide integration.

Moving forward, 5 areas require coordinated attention. First, larger-scale clinical and simulation trials must be launched to test UAV safety and efficacy under real-world conditions. Second, new airspace classifications should be introduced for medical-use drones, including provisions for emergency use. Third, legal and ethical frameworks must be clarified to ensure safety, liability, and consent standards are met. Fourth, UAVs must be integrated into existing logistics frameworks such as UNOS, NHS Blood and Transplant, and Eurotransplant. Finally, public confidence and clinical engagement must be cultivated through transparent communication and codesigned operational protocols.

With the right structures in place, drones could become a transformative part of transplant logistics, improving delivery speed, reducing costs and emissions, and ultimately helping more patients receive life-saving organs in time.
